# Effects and Mechanisms of Fruit and Vegetable Juices on Cardiovascular Diseases

**DOI:** 10.3390/ijms18030555

**Published:** 2017-03-04

**Authors:** Jie Zheng, Yue Zhou, Sha Li, Pei Zhang, Tong Zhou, Dong-Ping Xu, Hua-Bin Li

**Affiliations:** 1 Guangdong Provincial Key Laboratory of Food, Nutrition and Health, School of Public Health, Sun Yat-sen University, Guangzhou 510080, China; zhengj37@mail2.sysu.edu.cn (J.Z.); zhouyue3@mail2.sysu.edu.cn (Y.Z.); zhangp95@mail2.sysu.edu.cn (P.Z.); zhout43@mail2.sysu.edu.cn (T.Z.); xudp@mail2.sysu.edu.cn (D.-P.X.); 2 School of Chinese Medicine, The University of Hong Kong, Hong Kong, China; u3003781@connect.hku.hk; 3 South China Sea Bioresource Exploitation and Utilization Collaborative Innovation Center, Sun Yat-sen University, Guangzhou 510006, China

**Keywords:** fruit juice, vegetable juice, cardiovascular disease, blood pressure, cholesterol

## Abstract

Many studies have indicated that consumption of vegetables and fruits are positively related to lower incidence of several chronic noncommunicable diseases. Although composition of fruit and vegetable juices is different from that of the edible portion of fruits and vegetables, they contain polyphenols and vitamins from fruits and vegetables. Drinking vegetable and fruit juices is very popular in many countries, and also an efficient way to improve consumption of fruits and vegetables. The studies showed that fruit and vegetable juices affect cardiovascular risk factors, such as lowering blood pressure and improving blood lipid profiles. The main mechanisms of action included antioxidant effects, improvement of the aspects of the cardiovascular system, inhibition of platelet aggregation, anti-inflammatory effects, and prevention of hyperhomocysteinemia. Drinking juices might be a potential way to improve cardiovascular health, especially mixtures of juices because they contain a variety of polyphenols, vitamins, and minerals from different fruits and vegetables. This review summarizes recent studies on the effects of fruit and vegetable juices on indicators of cardiovascular disease, and special attention is paid to the mechanisms of action.

## 1. Introduction

Many studies have shown that some natural products possess high antioxidant activities, such as vegetables, fruits, cereals, mushrooms, microalgae, wild flowers, and medicinal plants [1,2,3,4,5,6,7,8,9,10,11], which could be employed to prevent or treat several chronic noncommunicable diseases caused by oxidative stress [12,13,14,15,16]. Epidemiological studies also revealed that consuming fruits and vegetables were negatively associated with chronic noncommunicable diseases. The nutritionists have recommended that the public should eat more vegetables and fruits. Drinking juice is an effective method to promote consumption of fruit and vegetables, and is very popular in many countries [17,18,19,20]. A large amount of evidence shows that juice is a part of balanced diet offering profound risk reduction of many diseases, such as cancers, neurodegenerative diseases, and cardiovascular diseases [18,21,22].

Cardiovascular disease includes disorders of the blood vessels and heart, such as hypertension, cerebrovascular disease, peripheral artery disease, coronary heart disease, rheumatic heart disease, heart failure, and congenital heart disease. Cardiovascular disease is one of the major causes of chronic noncommunicable deaths, usually associated with metabolic disorders, including obesity and diabetes. Hypertension and high cholesterol are two risk factors for sudden stroke or heart attack, and abnormal blood lipids are also significant risk factors for cardiovascular diseases. A healthy heart and circulation system could benefit from a balanced diet with adequate fruits and vegetables. Epidemiological evidence supports a significantly positive correlation between eating fruits and vegetables and cardiovascular health [23,24,25,26]. Several studies reported healthy dietary patterns containing adequate fruits and vegetables show good results with respect to blood pressure control [27,28,29], and some studies found that there was a positive association between consuming fruits and vegetables and blood lipids control [30,31,32,33,34]. In addition, the relationship between obesity and cardiovascular diseases should be taken into account because sugar-sweetened juices might contribute to obesity [35,36,37,38,39,40].

Although composition of fruit and vegetable juices is different from that of the edible portion of fruits and vegetables, they contain polyphenols, vitamins, and minerals from fruits and vegetables. In fact, the consumption of vegetable juice was confirmed to help reach the daily dietary vegetable intake recommendations consistent with the 2005 Dietary Guidelines and the Dietary Approaches to Stop Hypertension (DASH) diet [34], and drinking fruit juice was also an effective way of supplementing fruits. In this review, Web of Science, MEDLINE, PubMed, and EMBASE databases were searched with the keywords “juice” and “fruit or vegetable” for relevant papers published in English in peer-reviewed journals. This paper summarizes current knowledge and recent studies on the effects of fruit and vegetable juices on the cardiovascular system. Special attention is paid to the effects on blood pressure and lipids, and the mechanisms of action have been discussed.

## 2. Juices and Blood Pressure

Intake of raw fruits and vegetables has been associated with low incidence of hypertension [41,42,43]. Several epidemiological studies paid attention to the effect of consumption of juices on blood pressure (Table 1).

Several studies revealed the relationship between consumption of fruit juice and blood pressure. For example, juice of sweetie fruit (a hybrid between grapefruit and pummelo) possessed an anti-hypertensive effect. Diastolic blood pressure (DBP) was reduced more by the high-flavonoid sweetie juice than by the low-flavonoid sweetie juice (*p* = 0.04). The flavonoids naringin and naritutin in sweetie juice might be the active ingredients contributing to the anti-hypertensive effect [44]. Moreover, a study was conducted to determine the effects of fresh juice of pomegranate (*Punica granatum* L.) on blood pressure. Consuming pomegranate juice consumption induced significant reductions in DBP (*p* = 0.038) and systolic blood pressure (SBP) (*p* = 0.002) [45]. Particularly, a meta-analysis focusing on the effect of fruit juice on blood pressure and cholesterol in humans was reported. DBP was reduced via fruit juice consumption by 2.07 mmHg, but SBP was not significantly affected [46].

Several studies have indicated that consumption of vegetable juice may reduce blood pressure. High nitrate content in certain vegetables might be a source of cardiovascular protective nitric oxide. A dietary nitrate load (500 mL of beetroot juice) could reduce blood pressure (Δ_max_ −10.4/8 mmHg) approximately 3 h after ingestion, which was reciprocally associated with the nitrite concentration in plasma [47]. Two other studies focusing on the ingestion of nitrate-rich beetroot showed dose-dependently blood pressure lowering effects [48,49]. Another study confirmed that SBP was lowered significantly after drinking beetroot juice [50]. In addition to beetroot juice, rich in nitrate, other vegetable juices have also drawn wide attention. For instance, in a study, hypertensive subjects showed a remarkable decrease in blood pressure after the 12-week consumption of vegetable juice [34]. Furthermore, a pilot study was conducted to discover whether blood pressure was influenced by drinking fresh carrot juice, and results showed that SBP decreased (*p* = 0.06), but not DBP [51].

SBP and DBP were decreased significantly in prehypertensive and hypertensive subjects after consumption of the juice powder, consisting primarily of fruits and vegetables. This study supported the notion that the mixture of fruit and vegetable juices led to a decrease in blood pressure [52].

The anti-hypertensive effect of the juices may be related to bioactive components, such as polyphenols, vitamins, minerals, and nitrate (Table 1). It is thought that polyphenols are the main components in fruit juice to cause a decrease in blood pressure, while nitrate and minerals play an important role in the anti-hypertensive effect of vegetable juice.

## 3. Juices and Blood Lipids

Many studies have paid attention to the effects of fruit and vegetable juices on blood lipids (Table 2). For example, the relationship between orange juice consumption and cardiovascular disease was investigated. The results showed that total cholesterol (TC), apo B, low-density lipoprotein–cholesterol (LDL-C), and low-density lipoprotein / high-density lipoprotein (LDL/HDL) ratio were all significantly lowered by consuming orange juice, while the serum levels of homocysteine, high-density lipoprotein-cholesterol (HDL-C) and apo A-1 were not affected [64]. In another study, the effects of an acai berry-based juice blend on lipid profile were studied in a pilot study. Serum lipid profile (triglycerides, cholesterol, and its fractions) was substantially improved after the intervention. The improving blood lipids effects of the juice blend were mainly in relation to the high content of total polyphenol [65]. Additionally, the effects of cloudy apple juice with vitamin C on serum cholesterol were investigated. Total cholesterol showed a trend to decrease in the vitamin C-rich apple juice intervention period [66]. In another study, the effects of consuming cranberry juice cocktail on cardiometabolic outcomes were examined. The results showed levels of cholesterol tended to be lowered by a cranberry juice cocktail [67]. In addition, ingestion of lycopene-rich tomato decreased cholesterol. A study showed that serum levels of cholesterol were markedly reduced, while serum levels of adiponectin and triglyceride were increased by the intervention [68].

In the study of Houston et al. [52], HDL-C (*p* = 0.025) and apo A (*p* = 0.004) were remarkably decreased after two-year consumption of encapsulated juice powder containing the extract of different fruits and vegetables. In another study, the effects of a juice mixture of komatsuna and fresh fruit on serum cholesterol were investigated, and the results showed that serum TC and LDL-C were significantly reduced [69]. In addition, the effects of the fiber in fruits and vegetables in the diet were determined by Kelsay et al. [70]. When consuming the low fiber diet, the DBP of half of the men was 80 mmHg or more, and their DBP was significantly lower when consuming a high fiber diet. It was speculated that the DBP was probably affected by the fiber in the diet, so clear juices were less healthy than the whole fruits and vegetables.

However, there are a number of studies showing that consumption of juice does not decrease blood pressure or improve the blood lipid profiles. Several studies were conducted to assess the effects of clear apple juice, cloudy apple juices, squeezed orange juice, and grapefruit juice on blood pressure in healthy adults or adults with hypercholesterolemia and hypertriglyceridemia. No effect of any of these interventions was found on SBP or DBP [53,54,55]. In addition, two studies assessing the effects of vegetable juice showed that either beetroot juice or low sodium vegetable juice could not decrease blood pressure in individuals with type 2 diabetes or in people with metabolic syndrome [60,63]. Similarly, clear juice increased LDL-C without affecting HDL-C, and lipid panels were not affected after ingestion of orange juice or pomegranate juice [45,54,55]. In addition, according to a meta-analysis, consuming fruit juice showed no significant effects on TC, HDL-C, and LDL-C in adults [46]. Furthermore, the plasma cholesterol, triglycerides, apo A, apo B, LDL, and HDL were not affected by drinking fresh carrot juice [51].

## 4. Mechanisms of Action of Juice on Cardiovascular Diseases

The main mechanisms of action of juices on cardiovascular diseases included antioxidant effects, improvement of the aspects of cardiovascular system, inhibition of platelet aggregation, anti-inflammatory effects, and prevention of hyperhomocysteinemia.

### 4.1. Antioxidant Effects

Hypercholesterolemia is related to increased lipid peroxidation. Pomegranate juice drew wide attention because of the effects on lipid peroxidation. According to Betanzos-Cabrera et al. [73], LDL was protected by pomegranate polyphenols against cell-mediated oxidation. The oxidatively-modified LDL was reduced by pomegranate polyphenols, for the interaction of macrophages with LDL inhibited its oxidation by scavenging reactive oxygen species and reactive nitrogen species. In another study, serum paraoxonase activity was increased by pomegranate polyphenols, which led to the hydrolysis of lipid peroxides in oxidized lipoproteins and in atherosclerotic lesions. It was demonstrated that pomegranate polyphenols had antioxidant and anti-atherogenic effects both in vitro and in vivo in humans, and in atherosclerotic apo E deficient mice [60]. LDL oxidation was prevented by esterase paraoxonase1 (PON1) in animals. Cardiovascular disease was related to decreased levels of PON1. The effect of pomegranate juice on PON1 gene expression was investigated on streptozotocin-induced diabetic mice fed with a high-fat diet. Supplements with pomegranate juice significantly induced PON1 gene expression and activity in mice. In addition, blood glucose was dramatically reduced by the pomegranate juice, but not triacylglycerols and cholesterol levels [74]. Additionally, in the study of Potter et al. [51], the plasma total antioxidant capacity was significantly increased (*p* < 0.05) and the plasma malondialdehyde production was decreased (*p* < 0.05) after consuming 16 fl oz of fresh carrot juice daily for three months. Furthermore, in the study of Foroudi et al. [54], consuming orange juice markedly upregulated the total plasma antioxidant capacity, and remarkably reduced lipid peroxidation. In another study [75], after taking in an antioxidant-rich juice daily, plasma lipid oxidation (malondialdehyde concentration) was significantly decreased (−29%), while plasma antioxidant capacity was markedly increased (+115%) after the intervention. In another study, a commercial juice powder containing mangifera was investigated. The concentrations of ascorbic acid and plasma β-carotene were significantly increased after consuming the juice powder. Plasma total antioxidant status was also upregulated [76]. The antioxidant effect of green juice (orange, apple, lettuce, cabbage, and cucumber) was compared with orange juice in the study of Oliveira et al. [77]. The results showed that both green juice and orange juice exerted antioxidant effects, and green juice reduced weight gain, lipoperoxidation, and catalase activity. In another study, a juice made from dried apples and mandarin juice was investigated in obese children, and the antioxidant capacity of plasma showed a remarkable increase after the intake of the food, and DNA oxidative damage was reduced [78]. In the study of Huebbe et al. [79], inducible NO synthase was significantly inhibited by blackcurrant treatment in cultured macrophages and, compared with untreated controls, levels of iNOS protein were reduced while levels of heme oxygenase 1 were increased. In human subjects, blackcurrant significantly elevated both plasma ascorbic acid and radical-scavenging capacity. According to Miglio et al. [80], concomitant ingestion of antioxidant-rich fruit juice could prevent the increase in levels of uric acid and thiols induced by high-fat meals. In addition, the antioxidant defense mechanism against CCl_4_-induced reproductive toxicity could be augmented by pomegranate juice [81]. In addition, in an animal study [82], the hamsters received an atherogenic diet with either juice or tea daily. Aortic lipid deposition was inhibited after 12-week berry juices and teas, and reduced activity of hepatic antioxidant enzymes was triggered. It was suggested that the development of early atherosclerosis could be prevented by moderate consumption of berry juices. The composition and concentration of polyphenols in the juices were different, indicating that a diversity of polyphenols could induce anti-atherosclerotic effects.

However, some juices did not have antioxidant effects. Onion was rich in flavonoids, which were potential antioxidants. The effect of decocted onion juice (about 300 g onion) on mild hypercholesterolemic subjects was investigated. Twenty-seven subjects were recruited and the study lasted for 10 weeks. No significant differences were found in plasma total antioxidant capacity, antioxidant vitamins, LDL oxidation, or erythrocyte antioxidant enzyme activity [83]. In another study [53], the antioxidant effect of grapefruit was investigated, but results showed that oxidative stress was not affected after consumption of grapefruit juice.

### 4.2. Improving Aspects of the Cardiovascular System

Vascular health was affected by phenolic compounds of fruits and vegetables, and the cardiovascular protection of polyphenols was confirmed [16]. Endothelium-dependent vasodilatation played a key role in blood pressure. Poudyal et al. [84] compared the ability of purple carrot juice and β-carotene to reverse the structural and functional changes in the rat model of the metabolic syndrome. Endothelial dysfunction was observed in the rats. Both β-carotene and purple carrot juice were able to attenuate or reverse the endothelial dysfunction. It indicated that anthocyanins, as well as β-carotene, were related to improving the endothelial function. In addition, an animal study was developed to determine whether the blood pressure and vascular function of spontaneously hypertensive rats were affected by Finnish berry juices, including juices of lingonberry (*Vaccinium vitis-idaea*), cranberry (*Vaccinium oxycoccos*), and blackcurrant (*Ribes nigrum*) after the eight-week treatment. The results turned out that the impaired endothelium-dependent relaxation was observed in the cranberry, blackcurrant, and control groups, while it was normalized in the lingonberry group. All three kinds of fruit were rich in polyphenols. It could be concluded that endothelium-dependent vasodilatation of spontaneously hypertensive rats could be improved by long-term lingonberry juice treatment [85]. In another study, cranberry juice, which was rich in polyphenols, was investigated and no significant change caused by cranberry juice on peripheral endothelial function was found during the study [86]. Moreover, in the study of Asgary et al. [45], the effects of consuming juice of pomegranate on endothelial function were discussed. Serum levels of vascular endothelial adhesion molecule 1 (VCAM-1) were markedly down-regulated by pomegranate juice while those of E-selection were up-regulated. Additionally, in the study of Habauzit et al. [53], the effects of consuming grapefruit flavanone on vascular function were investigated. Central aortic stiffness was statistically significantly lowered after consumption of grapefruit juice. However, endothelial function in micro- and macrocirculation were not affected by the intervention. It could be concluded that grapefruit juice consumption was beneficial for arterial stiffness in middle-aged, healthy, postmenopausal women, which was related to flavanones presented in grapefruit. In the study of Auger et al. [87], the results indicated that very few commercial fruit juices could induce potent endothelium-dependent relaxations. The total polyphenol contents of active fruit juices and the less active juices were almost the same, so this effect did not contribute to their total polyphenol content, but to their specific polyphenol composition. In the study of Buscemi et al. [88], the effect of red orange juice consumption on endothelial function was investigated in subjects with higher cardiovascular risk. As a result, endothelial function was significantly improved. Additionally, the oriental plum (*Prunus mume*) fruit juice concentrate could improve human blood fluidity. Angiotensin II (AngII) stimulated the growth of vascular smooth muscle cells (VSMCs), through transactivating epidermal growth factor (EGF) receptor, which involved producing reactive oxygen species. Furthermore, the oriental plum fruit juice concentrate could inhibit AngII-induced EGF receptor transactivation. H_2_O_2_-induced EGF receptor transactivation was also inhibited. Thus, the oriental plum fruit juice concentrate remarkably inhibited AngII-induced extracellular signal-regulated kinase (ERK) activation. The oriental plum fruit juice concentrate inhibited AngII stimulated leucine uptake in VSMCs significantly. It was suggested that the oriental plum fruit juice concentrate could exert a significant cardiovascular protective effect [89]. Moreover, endothelial function and arterial tree vascular elastic properties could be improved by consuming Concord grape juice [90].

According to some studies [47,48,49,51,52], certain vegetables (such as beetroot) improved vascular protective NO endogenously, which could be elevated by ingestion of dietary nitrate by oral bacteria in the entero-salivary circulation. Consumption of beetroot juice containing nitrate might downregulate blood pressure, thus decreasing the risk of cardiovascular events. Blood pressure was lowered when consuming beetroot juice as part of a normal diet or supplement in most studies. Endothelial dysfunction was prevented by dietary nitrate. In addition, the underlying mechanism of *Aronia melanocarpa* (chokeberry) juice was determined. *A. melanocarpa* juice stimulated the endothelial formation of NO in coronary arteries; involving the phosphorylation of eNOS via activating the Src/PI3-kinase/Akt pathway [47,48,49,51,52].

The effects of blackcurrant juice on vascular function were investigated, and the flow-mediated dilatation increased significantly in the high blackcurrant juice drink group. Plasma vitamin C concentration was upregulated remarkably both in the low and high blackcurrant juice drink groups. In addition, the changes in plasma vitamin C and flow-mediated dilatation were correlative, remarkably. It could be concluded that vascular health was improved by consumption of blackcurrant juice resulting from elevating plasma vitamin C concentration [91]. Furthermore, results from the study of Noratto et al. [92] showed that plasma levels of MCP-1 and mRNA, as well as protein levels of VCAM-1, were lowered in the peach and plum groups. In another study, the effect of citrus fruits juice on vascular remodeling was assessed in a mouse model. Neointima formation was dramatically attenuated by 40% citrus unshiu juice, 10% citrus iyo juice, and 40% citrus iyo juice, but not 10% citrus unshiu juice. Vascular remodeling was attenuated partly through reducing oxidative stress [93]. In addition, in the study of Poudyal et al. [84], purple carrot juice was able to attenuate or reverse all of these changes. Carotenoid concentration was low in purple carrot juice, so anthocyanins might be the main contributor of antioxidant and anti-inflammatory properties to improve cardiovascular function. In the study of Ramli et al. [94], it was demonstrated that consuming red pitaya juice could reduce diastolic stiffness of the heart in rats. Aditionally, blood pressure downregulation effect of *Carica papaya* fruit juice was examined in male albino Wistar rats [95]. A significant depression of arterial blood pressure (MAP) was produced by juice and hydrallazine, and juice produced more depression of MAP than hydrallazine in the hypertensive rats. In vitro studies were conducted to explore the mechanism. Isolated rabbit arteries were used, and the results indicated that the juice produced relaxation of vascular muscle tone. It was concluded that *C*. *papaya* fruit juice could contain antihypertensive agents which exhibited mainly α-adrenoceptor activity.

### 4.3. Inhibitory Effect on Platelet Aggregation

Platelets were involved in atherosclerotic disease development and it was important to reduce platelet activity in the patients. Flavonoids could reduce platelet aggregation and lower the incidence of cardiovascular disease. Red wine and grapes were confirmed to be rich in polyphenolic compounds, including flavonoids. Citrus fruits contained different types of polyphenolics. In a randomized cross-over trial [96], the results turned out that the whole blood platelet aggregation responded to 1 mg/L of collagen was reduced by purple grape juice by 77% (from 17.9 ± 2.3 to 4.0 ± 6.8 ohms, *p* = 0.0002), but not affected by orange juice or grapefruit juice. The total polyphenolic concentration of purple grape juice was approximately two more times than that of the citrus juices. The flavonoids in grape juice reduced the risk of myocardial infarction and coronary thrombosis because of the platelet inhibitory effects. Another study [47] focused on the effects of beetroot juice consumption on platelet aggregation. The results showed that platelet aggregation was inhibited by the intervention. Additionally, in the study of Mattiello et al. [97], the antiplatelet effect of pomegranate (*Punica granatum*) juice was investigated. All of the platelet responses were reduced by pomegranate juices in study in vitro. The effective components were primarily hydrolyzed tannins, including ellagitannins. It could be concluded that cardiovascular health benefits of pomegranate were related to platelet inhibitory function of polyphenols.

The effect of consuming red or blond orange juice on whole blood procoagulant activity was investigated on healthy subjects. The red orange juice was rich in anthocyanins while the blond was not. The results showed that both intake of the two types of orange juice caused a prolongation of unstimulated and stimulated whole-blood clotting times, without any difference. It was suggested that procoagulant activity was decreased by orange juice, independent of its anthocyanin content [98].

### 4.4. Anti-Inflammation

Persistent inflammation was related to most chronic diseases including cardiovascular diseases [99]. Some diets reduced inflammation which was related to almost all phases of atherosclerosis, thus, they might reduce cardiovascular disease risk. The anti-inflammatory effects of some juices were confirmed in cardiovascular system. In the study of Li et al. [68], the effect of tomato juice rich in lycopene on monocyte chemoattractant protein-1 (MCP-1) was studied and the result showed that MCP-1 was markedly reduced after consumption of tomato juice containing lycopene. In addition, in the study of Noratto et al. [92], effects of plum and peach juice on obesity-induced inflammation related to cardiac dysfunction and heart failure were studied, and the results showed that protein levels of the active p-Iκ-Bα and p-NF-κBp65 subunits in heart tissue were lowered. In the plum juice group, mRNA levels of Iκ-Bα and TNF-α were significantly decreased. In the peach juice group, mRNA levels of TNF-α were lowered. In the study of Duffey and Sutherland [67], C-reactive protein was tested in cranberry juice cocktail consumers who consumed cranberry juice cocktail for two nonconsecutive 24-h dietary recalls. The results showed that C-reactive protein levels of adult cranberry juice cocktail consumers were significantly lowered. In the study of Codoner-Franch et al. [78], a novel food product containing mandarin juice was proved to reduce inflammatory markers of the subjects, such as tumor necrosis factor-α (TNF-α), high-sensitive C-reactive protein, and interleukins 1α and 6. In the study of Buscemi et al. [88], the effect of consuming red orange juice on inflammation markers in subjects with high cardiovascular risk was investigated. The results showed that high-sensitivity C-reactive protein, TNF-α, and IL-6 were remarkably downregulated.

However, some juices were not anti-inflammatory. In a placebo-controlled randomized study, orange juice or beverage alone did not lead to any remarkable effects on circulating cytokine levels or PAI-1 activity. However, IL-lβ and serum IL-6 levels were reduced significantly by sterol-fortified orange juice (*p* < 0.05) compared with baseline. No evidence has shown that orange juice alone could lower inflammatory biomarkers [100]. Additionally, in the study of Habauzit et al. [53], grapefruit juice was investigated. The result showed that biomarkers of inflammation were not affected by flavanones (naringenin glycosides) in grapefruit juice. Furthermore, in the study of Simao et al. [101], subjects with the metabolic syndrome consumed reduced-energy cranberry juice containing folic acid, after which the folic acid and adiponectin were increased (*p* = 0.010, *p* = 0.033, respectively), but the homocysteine was significantly decreased (*p* < 0.001). In another study, lipopolysaccharide-induced inflammation was significantly inhibited by blackcurrant treatment in cultured macrophages. Blackcurrant meal consumption did not remarkably change the production of TNF-α or IL-1β in the human subjects [79].

### 4.5. Preventing Hyperhomocysteinemia

Homocysteine drew attention in several studies. The effect of supplementation with fruit and vegetable juices on folate status, plasma homocysteine levels was determined in smokers, hypertensive patients, men with mild hypercholesterolemia, or patients with the metabolic syndrome. The results indicated that serum folate was increased and correlated with a decrease in plasma homocysteine [64,83,101,102,103,104].

### 4.6. Contributing to Body Weight Control

Some juices with low calorie content showed the potential prevent metabolic disorders, which is also beneficial for cardiovascular health. For example, mulberry (*Morus australis* Poir) and blueberry (*Vaccinium ashei*) juice was rich in anthocyanin, and it might reduce obesity. C57BL/6 mice were used to investigate the hypothesis. The results showed that body weight was reduced, serum cholesterol was down-regulated, and the resistance to insulin, lipid accumulation, and leptin secretin were inhibited in the high-fat diet mice [105]. In the study of Noratto et al. [92], results showed that taking in plum and peach juice could prevent metabolic disorders induced by obesity, and plum juice remarkably inhibited body weight gain. The content of polyphenols in plum was three times that of peach, which was related to the weight-reducing effect. In the study of Oliveira et al. [77], the mice taking in green juice as a supplement had remarkably less weight gain than the mice receiving water.

## 5. Possible Adverse Effects of Juices

It should be noted that the calorie content of juices might complicate the relationship between juice consumption and health. Some commercial fruit/vegetable juices contain too much sugar, which might increase the risk on weight gain/obesity and diabetes, and counteract the health benefits. According to the study of Morgan et al., drinking high-fructose corn syrup beverages could lead to childhood obesity, while limiting consumption of sweetened beverages may help decrease obesity in children [39]. In another study, consuming excess free fructose regularly was associated with arthritis in adults [106]. Additionally, consuming sugar-sweetened beverages regularly was associated with type 2 diabetes, and sweetened fruit juice was not a healthy alternative for preventing diabetes [40]. In addition, compared with solid foods, juices have a smaller satiating effect, so juice consumers might take in more energy. Regular excessive consumption of 100% fruit juice might contribute to weight gain/obesity because of the sugar naturally contained in fruits, so excessive consumption of juice is not recommended [107].

## 6. Conclusions

The effects of juices on cardiovascular diseases were widely studied. A large number of studies supported the view that consumption of juice could prevent the increase of blood pressure and improve lipids. Some juices, such as sweetie fruit juice, pomegranate juice, guava fruit juice, cherry juice, and beetroot juice could improve both SBP and DBP. On the other hand, juices like apple juice, berry juice, tomato juice could improve one’s lipid profile, such as lower serum LDL-C and total cholesterol, and increase adiponectin and triglyceride. The main underlying mechanisms of the cardiovascular protection included antioxidant effects, improvement of aspects of cardiovascular system, improvement of endothelial function, inhibition of platelet aggregation, anti-inflammation, and prevention of hyperhomocysteinemia. The effects of juices were related to components of the raw material, such as polyphenols and vitamins. The results suggested that some juices might be used as potential supplements for cardiovascular protection, especially mixture of juices containing a variety of fruits and vegetables with polyphenols, vitamins, and minerals. More epidemiological studies and further mechanism studies are still required to clarify the relationship between bioactive components, sugar and minerals in juices and cardiovascular health.

## Figures and Tables

**Table 1:** Effects of juices on blood pressure.

Juices	Effective Components	Subjects	Study Types	Results	Reference
Fruit juices					
Sweetie fruit juice	Naringin	Stage I hypertension	Cross-over	Decrease in systolic blood pressure (SBP) dose-dependent decrease in diastolic blood pressure (DBP)	[44]
Pomegranate juice	Ellagitannins and anthocyanins	Hypertensive	Randomized controlled	Decrease in SBP and DBP	[45]
Grapefruit juice	Flavanones	Healthy postmenopausal women	Randomized, controlled, crossover	No effect	[53]
Orange juice	-	Persons with hypercholesterolemia and hypertriglyceridemia	Randomized controlled	No effect	[54]
Clear and cloudy apple juices	Polyphenols	Healthy persons	Randomized crossover	No effect	[55]
Guava fruit juice	-	Healthy volunteers	Randomized, controlled	Decrease in SBP and DBP	[56]
Cherry juice	Anthocyanin	Healthy volunteers	Crossover	Decrease in SBP and DBP	[57]
Polyphenol-rich juices ^1^	Polyphenols	Healthy individuals	Randomized, controlled	Decrease in SBP and DBP	[58]
Purple grape juice	Anthocyanin	Smokers	Pilot	Decrease in DBP	[59]
Vegetable juices					
Commercial vegetable juice ^2^	Minerals and vitamins	Healthy persons	Randomized, controlled, parallel-arm	Decrease in BP	[34]
Beetroot juice	Nitrate	Healthy persons	Randomized crossover, Randomized, controlled	Decrease in BP	[47,48,49,50]
		Persons with type 2 diabetes	Randomized, crossover	No effect	[60]
		Hypertensive subjects	Randomized, crossover	Decrease in BP	[61]
		Hypertensive individuals	Randomized controlled	No effects	[62]
Carrot juice	Fiber, potassium, nitrates, and vitamin C	Persons with elevated Plasma cholesterol and Triglyceride levels	Pilot	Decrease in SBP	[51]
Commercial vegetable juice ^2^	Vitamin C and potassium	Persons with metabolic syndrome	Randomized, controlled, parallel-arm	No effects	[63]
Yam bean root juice	nitrate	Healthy volunteers	Randomized, controlled	Decrease in DBP	[56]
Mixture juice					
Fruit and vegetable powder juice ^3^	-	Healthy persons	Pilot	Decrease in SBP and DBP	[52]

^1^ Juice based on cherries, chokeberries, red grapes, bilberries, and blackcurrant; ^2^ Commercial vegetable juice (V8^®^; Campbell Soup Company, Camden, NJ, USA) that provided Vitamin A, Vitamin C, calcium and iron; ^3^ Encapsulated juice powder containing acerola cherry, apple, beet, tomato, etc.

**Table 2:** Effects of juices on blood lipids.

Juices	Effective Components	Subjects	Study Types	Results	Reference
Fruit juices					
Pomegranate juice	Ellagitannins and anthocyanins	Hypertensive	Randomized controlled	No effect	[45]
Orange juice	Vitamin C, folate, and potassium	Persons with hypercholesterolemia and hypertriglyceridemia	Randomized controlled	No effect	[54]
		Persons with normal and moderately high cholesterol blood levels	Cross-sectional	TC, LDL-C, apo B and LDL/HDL ratio were all significantly lowered ^1^	[64]
Apple juice	Polyphenols	Healthy persons	Randomized crossover	Lower serum LDL-C for cloudy juice, no effect for clear juice	[55]
	Vitamin C	Healthy persons	Randomized crossover	Decreased trend in total cholesterol	[66]
Acai berry juice	Polyphenols	Junior hurdlers	Pilot	Improvement in lipid profile	[65]
Cranberry juice	Polyphenols	Healthy persons	Cross-sectional, association	A tendency of lower levels of cholesterol	[67]
Chokeberry juice	-	Healthy volunteers	Pilot	No significant effect	[71]
Vegetable juices					
Carrot juice	Carotenoid, anthocyanin	Healthy persons	Pilot	No effect	[51]
Tomato juice	Lycopene, minerals	Healthy persons	Randomized controlled	Decrease in serum cholestol, increase in adiponectin and triglyceride	[68]
Tree tomato juice	-	Persons with hypercholesterolemia	Randomized, controlled	Decrease in TC and LDL-C	[72]
Mixture juices					
Mixture of vegetables and fruits juice ^2^	Minerals, vitamins, and polyphenols	Prehypertensive and hypertensive	Pilot	Decrease in HDL-C and apo A	[52]
Fruit and komatsuna juice ^3^	Minerals, vitamins. and polyphenols	Healthy persons	Randomized controlled	Decrease in TC and LDL-C	[69]

^1^ TC, total cholesterol; LDL-C, low-density lipoprotein–cholesterol; LDL/HDL ratio, low-density lipoprotein/high-density lipoprotein ratio; ^2^ It an encapsulated juice powder containing acerola cherry, apple, beet, tomato, etc.; ^3^ The fruit includes banana and apple.
